# Attention decouples action potentials from the phase of local field potentials in macaque visual cortical area MT

**DOI:** 10.1186/s12915-018-0551-2

**Published:** 2018-08-06

**Authors:** Moein Esghaei, Mohammad Reza Daliri, Stefan Treue

**Affiliations:** 1 Cognitive Neurobiology Laboratory, School of Cognitive Sciences, Institute for Research in Fundamental Sciences (IPM), Niavaran, P.O. Box 19395-5746, Tehran, Iran; 2 Biomedical Engineering Department, School of Electrical Engineering, Iran University of Science and Technology (IUST), Narmak, Tehran, 16846-13114 Iran; 3 Cognitive Neuroscience Laboratory, German Primate Center – Leibniz Institute for Primate Research, Goettingen, Germany; 4 Faculty of Biology and Psychology, University of Goettingen, Goettingen, Germany; 5 Bernstein Center for Computational Neuroscience, Goettingen, Germany; 6 Leibniz-ScienceCampus Primate Cognition, Goettingen, Germany

**Keywords:** Local field potential (LFP), Spike-phase coupling, Area MT, Macaque, Spatial attention, Inter-neuronal correlation, Delta, Theta

## Abstract

**Background:**

The timing of action potentials (“spikes”) of cortical neurons has been shown to be aligned to the phase of low-frequency (< 10 Hz) local field potentials (LFPs) in several cortical areas. However, across the areas, this alignment varies and the role of this spike-phase coupling (SPC) in cognitive functions is not well understood.

**Results:**

Here, we propose a role in the coordination of neural activity by selective attention. After refining previous analytical methods for measuring SPC, we show that first, SPC is present along the dorsal processing pathway in macaque visual cortex (area MT); second, spikes occur in falling phases of the low-frequency LFP independent of the location of spatial attention; third, switching spatial attention into the receptive field (RF) of MT neurons decreases this coupling; and finally, the LFP phase causally influences the spikes.

**Conclusions:**

Here, we show that spikes are coupled to the phase of low-frequency LFP along the dorsal visual pathway. Our data suggest that attention harnesses this spike-LFP coupling to de-synchronize neurons and thereby enhance the neural representation of the attended stimuli.

## Background

The frequency of neuronal impulses is the nervous system’s central principle for representing sensory stimuli. For instance, a large majority of neurons in the area MT of extrastriate primate visual cortex are direction-selective, i.e., they systematically vary their firing rate as a function of the direction of visual motion shown in their receptive fields [1, 2]. The local activity of neural populations creates local field potential, continuous periodic signals across a broad frequency spectrum, in contrast to the binary action potentials (“spikes”) generated by the individual neurons. Local field potentials (LFPs) represent mainly the synaptic activity from volumes within several hundred micrometers around the recording electrode [3, 4]. Higher frequency components of LFPs represent more local activity [5–7], while the activity in the lower frequency bands reflects the integration of neural activity across larger networks of neurons [8]. Although spike and LFP signals are often studied independently, it has also been shown that neuronal spike times are correlated with the phase of low-frequency (< 10 Hz) LFPs in a number of cortical areas including the primary visual [7] and the prefrontal cortex [9]. Similarly, Sirota et al. found for several cortical regions that local cortical spikes are coupled to the LFPs of the hippocampus in the theta band (3–5 Hz) [10]. This spike-phase coupling (SPC) might provide a mechanism for the coordination of the spike timing across the cortex that is thought to underlie cognitive functions, such as selective attention.

Visual attention is a mechanism for the selective allocation of processing resources to the most behaviorally relevant information [11–13] within the incoming stream of sensory signals. Converging evidence suggests that when spatial locations, features, or objects are attended, neurons in the visual cortex that represent them respond at higher spike rates [14–16]. For example, neurons in the macaque extrastriate visual cortex increase their spike rate when the animal’s spatial attention overlaps a given neuron’s receptive field (RF) [17]. Similarly, attention modulates low-frequency (< 10 Hz) local field potentials (LFPs) across different regions of the macaque cortex [18]. Specifically, visual attention decreases the power of low-frequency LFPs in macaque V4 [19, 20] and V1 [21]. Furthermore, Lakatos et al. reported that attention can shift the phase of low-frequency delta band (0–4 Hz) activity elicited by rhythmic stimulus presentations such that the phases of the delta band are better aligned with the stimulus presentations [22]. Together with the observation that attention may sample stimuli rhythmically at the rate of 4–10 Hz [23], these findings suggest a functional role of low-frequency oscillations in sensory processing and its attentional modulation.

The previously reported alignment of spike times to the phase of the low-frequency components of LFPs [7, 24] provides a potential mechanism for how low-frequency activity could affect the spike-based encoding of sensory information [25]. Given the observation that spiking activity across neighboring neurons shows an attentional decrease of their synchrony and their trial-by-trial correlations [26, 27], attention may use the spike-LFP coupling to influence inter-neuronal correlations.

Here, we hypothesize that the mechanism of this attentional de-synchronization could be mediated by de-coupling spiking activities from low-frequency synaptic activity (reflected in LFPs). To test this proposal, we trained rhesus monkeys to attend to a stimulus within the receptive field of MT neurons or to another stimulus outside the receptive field. We developed a refined method for calculating the SPC, avoiding the biases to differences of LFP power across time present in previous approaches. In support of our hypothesis, the data show that SPC decreases when spatial attention is allocated inside a receptive field.

## Methods

### Animal welfare

The scientists in this study are aware and are committed to the great responsibility they have in ensuring the best possible science with the least possible harm to any animals used in the scientific research [28]. All animal procedures of this study have been approved by the responsible regional government office (Niedersaechsisches Landesamt fuer Verbraucherschutz und Lebensmittelsicherheit (LAVES)) under the permit numbers 33.42502/08-07.02 and 33.14.42502-04-064/07. The animals were group-housed with other macaque monkeys in the facilities of the German Primate Center in Goettingen, Germany, in accordance with all applicable German and European regulations. The facility provides the animals with an enriched environment (including a multitude of toys and wooden structures [29, 30]) and natural as well as artificial light, exceeding the size requirements of the European regulations, including access to outdoor space. Surgeries were performed aseptically under gas anesthesia using standard techniques, including appropriate peri-surgical analgesia and monitoring to minimize potential suffering [31]. The German Primate Center has several staff veterinarians that regularly monitor and examine the animals and consult on procedures. During the study, the animals had unrestricted access to food and fluid, except on the days where the data were collected or the animals were trained on the behavioral paradigm. On these days, the animals were allowed unlimited access to fluid through their performance in the behavioral paradigm. Here, the animals received fluid rewards for every correctly performed trial. Throughout the study, the animals’ psychological and veterinary welfare was monitored by the veterinarians, the animal facility staff and the lab’s scientists, all specialized in working with non-human primates. The three animals participating in this study were healthy at the conclusion of our study and were subsequently used in other studies.

### Behavioral task and recording

**Fig. 1:**
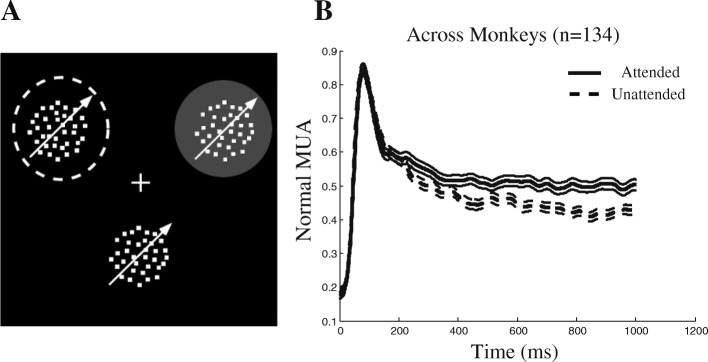
Behavioral paradigm to study spatial attention and attentional effects on neural activity. **a** Three monkeys were trained to covertly attend to a cued RDP while a single stimulus was presented inside the receptive field (RF, gray-filled circle) and one/two RDPs were presented outside the RF (one for monkey H and two for monkeys C and T). For illustration purposes, the fixation point is shown as a plus sign, and the target RDP has been marked with a white dashed-line circle. The target was cued using either a static RDP in the same position as the target (for monkey H) or a small rectangle beside the fixation point (for monkeys C and T). **b** Spike density function for the multi-unit activity (MUA) averaged across all recording sites starting from stimulus onset. The solid line represents the MUA when the target was inside the RF and the dashed line when the target was outside the RF. Error bars represent the standard error of the mean (SEM)

Three male macaque monkeys were trained to foveate a central fixation point and covertly attend to one of two or three coherently moving random dot patterns (RDPs) presented simultaneously at peripheral locations (Fig. 1a). A trial started after the monkey touched a lever and started foveating on the fixation point. Next, a spatial cue specified where the monkey had to attend to. For monkey H, this cue was a static RDP in the same position as the upcoming target stimulus, shown for 455 ms. For monkeys C and T, the cue was a small rectangle placed on a virtual line connecting the fixation point to the upcoming target, shown for 250 ms. The moving RDPs were presented after a brief delay of 325 ms (monkey H) or 500 ms (monkeys C and T) after the cue had disappeared, and the target changed its direction at a random time between 26 and 4250 ms after the RDPs’ onset. The monkeys were rewarded if they released the lever within a time window of 150–650 ms (monkey H) or 60–700 ms (monkeys C and T) after the direction change of the target. One of the RDPs was placed inside the receptive field (RF) and the other(s) outside. The RDP that was inside the RF and/or was targeted by the cue moved in the preferred direction of the neuron. The preferred direction of each neuron was determined using the direction tuning function [1] that was measured in the beginning of each session. The monkeys H, C, and T responded correctly to the direction change of the target in 86, 71, and 83%, respectively, of trials that were completed without fixation breaks.

Multi-unit activity (MUA) and LFP signals were recorded from the area MT using a five-channel recording system (MiniMatrix; Thomas Recording), using the guide tube as the reference electrode. Each electrode’s signal was split into LFP and spike components by hardware filters. The LFPs were amplified and digitized at 1 kHz, while spikes were amplified and digitized at 40 kHz. We recorded from up to all five electrodes (impedance 2 MΩ, arranged linearly and separated by 305 μm) simultaneously. Spikes were sorted online using the Plexon Data Acquisition System and next down sampled to 1 kHz. We selected sites according to the selectivity of the isolated cells to motion direction and the position of the electrode in the cortex using MRI images. In sessions with simultaneous recordings, we made sure that the RFs of the different units overlap sufficiently for all RFs to contain the stimulus. Only the sites with at least three trials in each attention condition were selected for the analyses, resulting in an average of ten trials per attention condition. Overall, we selected 326 site pairs from monkey H, 28 pairs from monkey C, and 28 pairs from monkey T (taking the spike from one electrode and LFPs from the other simultaneously recording electrodes) for our analysis (for more details on the task and recordings, see [32] for monkeys C and T and [33] for monkey H).

### Data analysis

We conducted the analyses in MATLAB (Mathworks, Natick, MA). To create the spike density function, we convolved a Gaussian of SD = 15 ms with the MUA spike trains [32] and normalized the result for each site by the maximum value across both attention conditions. We found the starting point of attention modulation on MUA by searching for the first ten consecutive time points (ms) that were significantly (*p* < 0.01 paired *t* test) different in firing rate between the two attention conditions. The attentional modulation index (AI) was calculated using the formula:

$$ \mathrm{AI}=\left({r}_{\mathrm{att}}-{r}_{\mathrm{unatt}}\right)/\left({r}_{\mathrm{att}}+{r}_{\mathrm{unatt}}\right), $$

where *r*_att_ and *r*_unatt_ correspond to the MUA response of each site in the attended and unattended conditions, respectively [34].

Before analyzing the LFPs, we aligned the LFP phases using the method proposed by Nelson et al. in order to correct the phase shifts created by the recording system [35]. All LFP signals were then normalized to their mean and std., so their average power and standard deviation would be equal. In order to filter the LFP signals into different low-frequency bands, we used EEGLAB toolbox’s *eegfilt* function [36] with the filter order of 3 × (sampling_rate/low_cutoff_freq) and assuming each trial’s LFP signal as one epoch (rather than applying independent filters on different sections of the signal). For each LFP signal, we tried different frequency bands with their starting frequencies changing between 1 and 15 Hz and bandwidths of 4 Hz. To calculate the spike-phase coupling (SPC) strength for each recorded spike-LFP pair, we first equalized the firing rate between the attended and unattended conditions. This is because the lower spike rate within a given interval is, the larger phase-aligned the spikes would be. This follows the same logic as when the difference between the proportion of the heads and tails for flipping a coin correlates negatively with the number of flips (imagine the number of flips as the number of spikes and the two falling conditions (head/tails) as two anti LFP phases) (see [37]; Fig. 4 for a simulation). We equalized the mean firing rate of each site across the two attention conditions by randomly removing the spikes from each spike train recorded at each trial. We then used the following equation:

$$ M=\frac{1}{n}\sum \limits_{k=1}^n{e}^{i{\varphi}_k}, $$

where *n* is the number of spikes in each spike train, *φ*_*k*_ denotes the instantaneous phase of the low-frequency band determined by Hilbert transform at the same time as the *k*th spike, and ${e}^{i{\varphi}_k}$ determines the complex exponential function of *φ*_*k*_ (following Euler’s formula). An estimation of the strength of spike-phase coupling is given by the length of vector M (|M|) (also known as the phase-locking value (PLV) [37]), and the locking phase of each pair is given by the angle of vector M. Vector M is long when most spikes are locked to a specific phase and short when spikes are not locked to a fixed phase across different trials. However, PLV depends on the number of spikes, and it also does not show how comparable it is for the neurons with different spike rates. In order to quantify the extent to which the spikes are coupled to the field potentials, we had to compare PLV with its distribution for randomly generated spike trains with the same rate as the original spike train. Therefore, for each trial, we generated 100 surrogate spike trains by randomly assigning spike times uniformly distributed in time and then calculated PLV for each surrogate spike train given the LFP signal filtered to the given frequency band. This gave us an estimation of the distribution of PLVs for the random spike trains with the same number of spikes as the original data and the filtered LFP. The PLV of the original trial was then *z*-scored to the mean and standard deviation of the corresponding distribution. We defined the resulting value as the *SPC index*. On the other hand, the distribution of instantaneous LFP phases within a given frequency band in each trial was not uniform (i.e., a trial’s LFP could have more rising parts rather than falling parts); therefore, we made it uniform using the following steps: for each trial, we partitioned the trial’s time points (1–600 ms aligned to 250 ms after the stimulus onset; sampling frequency 1000 Hz) based on their corresponding phases, into 30 equally wide phase bins ([− *π*, − *π* + 2*π*/30], [− *π* + 2*π*/30, − π + 4*π*/30], …, [*π* − 2*π*/30, *π*]). Next, an equal number of time points (as much as the mean number of time points in each phase bin) was randomly chosen from each phase bin (without the removal of time points from bins), providing a sequence of time points with the same length as the original signal. This selection was carried out independent of spike times. Those spikes corresponding to the sampled time points, together with their LFP phases, were extracted from the original pair of signals, and the SPC was calculated for them as explained above. This process was repeated 50 times for each trial, and the mean SPC value was taken as the SPC index for that trial.

SPC indices calculated using the above steps could be close to zero, both when the spikes are mostly locked to two (bipolar) or more (multi-polar) specific phases equally distanced around the circle, or when they are distributed uniformly around the circle. In both of these cases, the length of the averaged vector will not vary depending on the distribution of random vector lengths. Our measure does not distinguish between the bipolar (or multi-polar) case and the uniformly distributed case; this is because we aimed at identifying the condition where the spikes are locked to one specific phase.

To test if the SPC difference across attention conditions is not due to a difference in the signal-to-noise ratio of low-frequency LFP, we focused on a subset of trials that reflect an opposite effect of attention on low-frequency power, i.e., where attended trials have a higher mean low-frequency power compared to unattended trials (rather than the opposite, reported previously—see the “Results” section). To this end, we selected those attended trials where the 2–6 Hz (within which attention maximally modulates SPC) power was larger than the 4th percentile of the 2–6 Hz power distribution in the unattended trials. Similarly, for the unattended trials, we selected those trials where the 2–6 Hz power was in the 96th percentile of the 2–6 Hz power distribution in the attended trials. This gave us a set of trials for each attention condition, where all attended trials had a low-frequency power higher than that of the unattended trials (rather than unattended trials larger than attended trials).

To evaluate the correlation between attentional modulations of the spike rate and SPC, we considered the data from all three animals. To calculate the SPC index of each site, we averaged the SPC index calculated between that site’s spikes and the LFP recorded simultaneously from each of the other sites, for each attention condition separately. We next calculated the correlation between the difference of the resultant SPC index across attention conditions and the attentional modulation of the spike rate (AI) through sites, separately for each of the frequency bands (as in Fig. 3).

To control for the effect of volume conductance, we subtracted from each site’s LFP the average across all other available sites’ LFPs of the same time point, except that of the spike-providing site (to exclude any contamination of the potential leakage of the spike waveforms into the recorded LFP in calculating the SPC index). This allowed us to address those LFP components which are localized to the LFP-providing site. This analysis included 372 site pairs across animals (a number of site pairs reflected in Fig. 3 were not considered due to those sessions with only two parallel recordings, limiting any average referencing). This analysis was performed for the 4-Hz-wide frequency bands with their centers between 3 and 8 Hz.

To calculate the locking phase for each site pair, vector M was computed for each trial, and then the mean vector was calculated across the attended and unattended trials separately. The phase of the given vector for each condition was taken as the locking phase. To correct for multiple comparisons, we used the Bonferroni correction throughout.

## Results

Three rhesus monkeys were trained to direct their covert spatial attention towards a cued eccentric position on a computer screen while maintaining their gaze on a central fixation point. They were rewarded for reporting a small direction change in the moving random dot pattern (RDP) presented at the cued location (target stimulus) while ignoring the other stimuli presented at a location in the opposite visual hemifield (the distracters). The stimulus inside the RF was the target in half of the trials (attended condition) and the distracter in the other half (unattended condition) (Fig. 1a). To validate whether the monkeys actually attended selectively to the cued positions, we compared the neural activity across the different attention conditions. Figure 1b plots the spike density functions of the multi-unit activity (MUA) for the three monkeys from the onset of the target for the attended and the unattended condition. After about 245 ms, the activity in the attended condition becomes significantly greater than the unattended condition (see the “Methods” section for details) for the remainder of the trial. For the period from 250 to 850 ms after stimulus onset, the response in the attended condition was on average 10% higher than the unattended condition (see the “Methods” section for details). This confirms the well-known modulation of MT neural responses by spatial attention.

### Spike-phase coupling is suppressed by attention

**Fig. 2:**
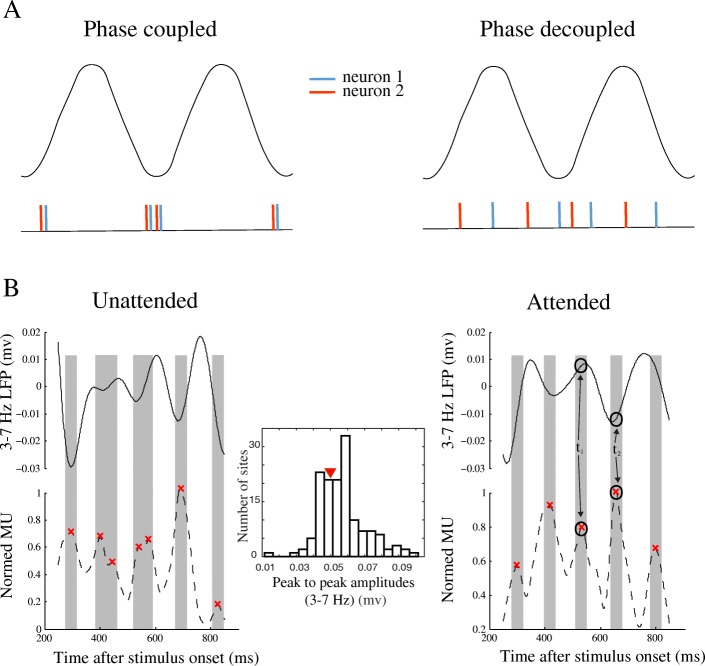
Example of spike-phase coupling (SPC). **a** An illustration of how SPC leads to inter-neuronal synchrony. Oscillatory curves on the top illustrate two cycles of a hypothetical LFP signal in a narrow frequency band. The spiking activity of two example neurons (artificial data) is shown in red and blue in two synchronized (spikes coupled to the LFP trough) and desynchronized (spikes distributed across LFP phases) conditions. **b** Sample trial data. MUA and LFPs, filtered to extract theta band (3–7 Hz) activity, in an example attended (right panel) and unattended (left panel) trial of a sample site from monkey H. Middle panel presents where the peak-to-peak amplitude of this sample site’s LFP (within 3–7 Hz) stands (shown by the red triangle) over the histogram of the peak to peaks across all sites recorded from. The peaks of the MUA and a representative neighboring time window around them are marked by the red crosses and gray bars, respectively. Note that the sample site presented here does not necessarily follow the population’s average preferred phase (see Fig. 6)

It is well established that attention modulates inter-neuronal synchronization; however, the neural mechanism is not well-understood. Here, we suggest that this modulation is implemented by controlling how strong the spikes are coupled to the instantaneous state of the population synaptic activity (represented by the phase of the LFP). Figure 2a shows how a stronger coupling of spikes to LFP phase leads to a higher inter-neuronal synchrony. The left panel illustrates the spike train of two example neurons (each neuron shown in one color), where both neurons are coupled to the trough of the LFP. As shown in the lower panel, the two neurons fire with a high synchrony. On the other hand, when the two neurons are not coupled to the LFP phase, generating spikes at different phases of the LFP (Fig. 2a, right panel), the spikes from the two neurons occur independently of each other. This suggests that a higher spike-phase coupling (SPC) is a signature of a higher inter-neuronal synchrony.

To test if attention influences SPC, we analyzed the steady-state component of the neural responses, starting 250 ms after the stimulus onset, to avoid the stimulus-locked low-frequency component of LFP. The data of a sample site recorded from monkey H are presented in Fig. 2b. The left panel shows the multi-unit activity (MUA, bottom) and the local field potentials (LFP, top) for a sample trial in the unattended condition, and the right panel shows the same signals for a sample trial in the attended condition. The MUA signals are smoothed and normalized to their maximum, and the LFPs are filtered to 3–7 Hz (theta band). It is clear in the left panel of this example that the peaks of the MUA are coupled to the phase of the low-frequency LFP in the unattended condition. However, in the right panel, we observe a MUA peak in *t*_1_ that corresponds to a peak of the LFP (as marked with circles), while another MUA peak at *t*_2_ occurred with an LFP trough. Therefore, in the attended condition, the MUA peaks show a reduced coupling to the LFP signal, i.e., MUA peaks occur at arbitrary phases of the LFP.

To verify the generality of the effect observed at the example site, we calculated the strength of spike-phase coupling (SPC) between the recorded MUA spikes and the recorded LFPs at low frequency ranges. In order to control any potential spectral leakage of spikes on low-frequency LFPs, we chose LFPs and MUAs from separate sites recorded at the same time. SPC strength was calculated by first assigning a vector of length 1 to each spike with the same phase as the LFP point corresponding to the spike time and next computing the mean vector across all vectors corresponding to a given trial (see the “Methods” section for details). The SPC was determined for the time interval of 250–850 ms after stimulus onset. In order to avoid any possible effects of spike rate modulation by attention, the average spike rates in the attended and unattended conditions were equalized across sites by randomly removing the spikes. An index (SPC index) was used to quantify the coupling strength by comparing the SPC strength for the real signal with the SPC strength of a random signal (see the “Methods” section for details).

**Fig. 3:**
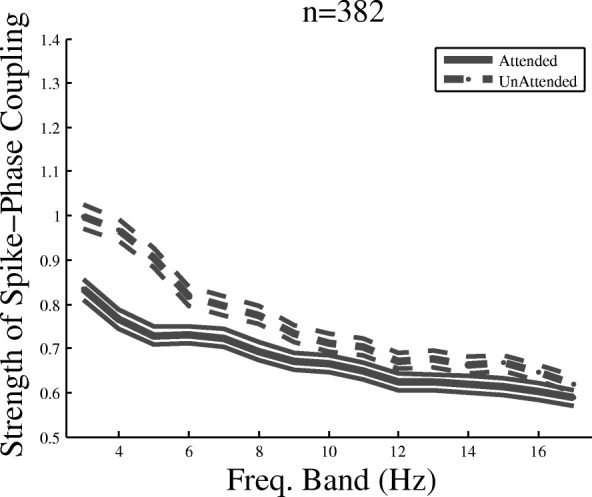
Spike-phase coupling (SPC) index at different low-frequency bands for the two attention conditions. The *x*-axis shows the middle frequency of 4 Hz windows stepped by 1 Hz, and *y*-axis shows the SPC index corresponding to each frequency band averaged across the population of site pairs across monkeys. The attended condition data are shown using solid lines, and the unattended condition is shown using dashed lines. The number of site pairs is provided on the top of the figure. Error bars are SEM

Next, we filtered the LFPs into overlapping 4 Hz windows stepped by 1 Hz. For each frequency window, we calculated the SPC index in the two attention conditions. Figure 3 shows the SPC index for all pairs of sites recorded from the three monkeys in the two attention conditions across different frequency bands. The *x*-axis represents the center of the frequency bands, and the *y*-axis reflects the corresponding value of the SPC index. The SPC index is significantly above zero for both attended and unattended conditions in all 3–17 Hz frequency bands (*p* ≪  0.001 *t* test, *p* > 0.1 Kolmogorov-Smirnov test for each bin, corrected for multiple comparisons). This shows that MUA spikes are locked to specific phases of the LFP within frequencies below 18 Hz independently of the attention condition. The SPC index in the unattended condition is significantly larger than the attended condition for those frequency bands that are centered between 3 and 8 Hz (17%, *p* < 0.01 paired *t* test, corrected for multiple comparisons). This indicates that attention is correlated with a decrease of SPC in the frequency bands below approximately 7 Hz. The same analyses conducted on each monkey’s data separately showed a similar effect within the 3–8 Hz frequency range, with 19, 17, and 6% higher SPC index in the unattended compared to the attended condition for monkeys H, C, and T, respectively.

One concern might be that the SPC difference between the two attention conditions is due to the difference between the power of low-frequency LFP in the attended and unattended condition. Since attention suppresses the power of low-frequency LFP oscillations (shown by us [38] and others [19]), this might reduce the signal-to-noise ratio of the low-frequency LFP in the attended condition and therefore the accuracy of the phase estimation, compared to the unattended condition. To test this, we focused on a subset of trials that reflect an opposite effect of attention on low-frequency power, i.e., where attended trials have a higher mean low-frequency power compared to unattended trials. This analysis removes a considerable number of trials; therefore, we focused on monkey H’s data, where the animal performed enough number of trials. For each site, we selected those trials which lead to an enhancement of LFP power in the 2–6 Hz band (which undergoes the maximum SPC modulation) compared to the unattended condition averaged across trials with all motion directions (see the “Methods” section for details). The data show a statistically significant enhancement of LFP power in the attended condition (*p* < 10^−6^; sign test across sites). Comparing the SPC index using the above approach based on this selection of trials again led to an enhancement of the SPC index in the unattended compared to the attended condition (15%, *p* < 0.01; paired *t* test across site pairs).

Furthermore, it could be argued that the suppression of SPC in the attended condition is simply induced by the increase of firing rate by attention. Assuming a given neuron’s membrane potential that oscillates within a low-frequency band and only a mild voltage input to the neuron, the cell is able to generate an action potential only in a small time window around the high-excitability phase of its membrane oscillation. Obviously, an enhancement in the neuron’s input voltage allows the cell membrane to reach the firing threshold voltage within a longer time window, consequently allowing the spikes to fire in a wider range of phases (equivalent to a suppression of SPC). To evaluate if the decrease of SPC that we observed due to attention is a side effect of the attentional enhancement of firing rate, we computed the attentional modulation of SPC (unattended SPC index − attended SPC index) and the attentional modulation of spike rate (AI) (see the “Methods” section for details) for different recording sites. The correlation of the two parameters was next computed across sites for each of the frequency bands (as described in Fig. 3). This showed no significant correlation between the attentional modulation of spike rates and SPC index, within any of the frequency bands (Spearman *R* = 0.04 ± 0.08 (SD), *p* > 0.05). This suggests not only that the attentional suppression of SPC observed here is not caused by an increase of spike rate by attention but also that the neural mechanism underlying this attentional modulation of SPC is distinct from that of the attentional enhancement of spike rate (see the “Discussion” section).

**Fig. 4:**
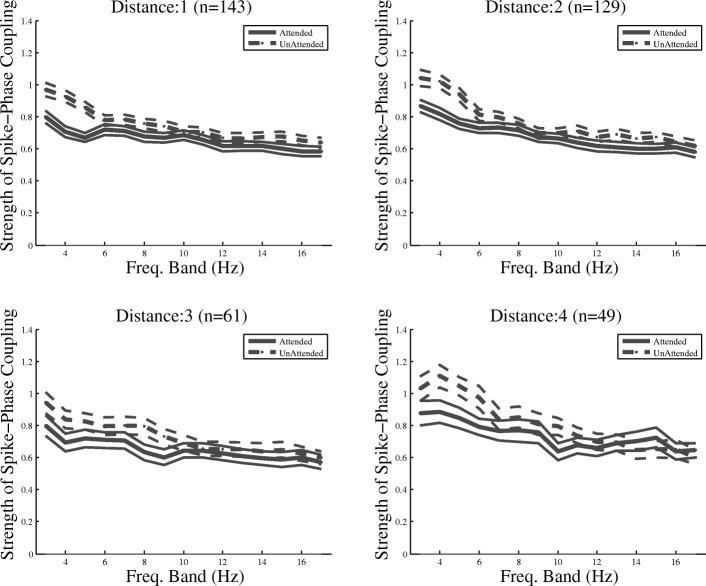
SPC index at different low-frequency bands plotted separately for site pairs with an equal number of recording sites in between. The distance between the pairs and the number of site pairs for each distance is given above each figure

To determine if the modulation of SPC is limited to only a specific distance between site pairs that provide the LFP and MUA, similar analyses were conducted on pairs of sites with equal horizontal distances in between, i.e., pairs of sites with one electrode in between, pairs with two electrodes in between, etc. Across all distances between the site pairs, SPC modulation could be found throughout the frequency bands with center frequencies between 3 and 8 Hz (Fig. 4), similar to Fig. 3 (for data pooled across distances), indicating that SPC is modulated within the electrodes as close as 305 μm to as distant as 1220 μm. These results suggest that attention widely suppresses the coupling between spikes and low-frequency LFPs in delta (1–4 Hz) and theta (4–8 Hz) bands in MT, consistent with the previous findings in other visual areas [39].

**Fig. 5:**
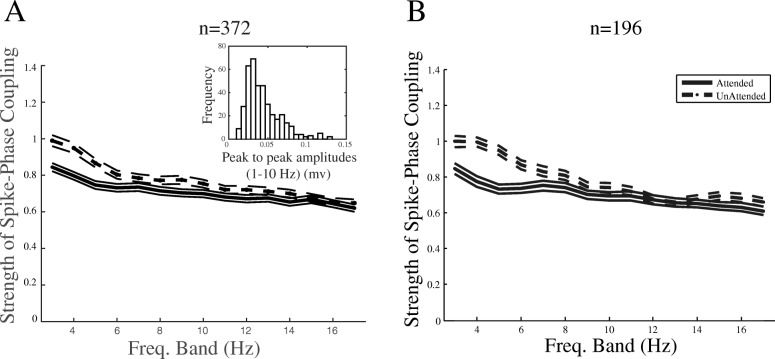
Attentional modulation of SPC is local and confined to delta and theta bands. **a** SPC index after controlling for volume conductance. We subtracted from each site’s LFP the average LFP recorded from the neighboring sites. Using these localized LFPs, we calculated the SPC index across attention conditions for each of the 4-Hz wide frequency bands, with their centers shown on the *x*-axis. The inset shows the peak-to-peak LFP amplitude within the frequencies lower than 10 Hz. **b** SPC index at different low-frequency bands for the two attention conditions for pairs of sites with at least ten trials in each condition

To further validate if the low-frequency component that coordinates the spikes originates within the area MT’s local population, rather than a larger and possibly extended structure elsewhere (transmitted by volume conductance), we subtracted from each site’s LFP the average LFP recorded from the neighboring sites. Using these *localized* LFPs, we calculated the SPC index across attention conditions for each of the frequency bands within the range over which we have observed an attentional modulation (3–8 Hz). This led to a significant difference between the SPC indices across attention conditions for all of the above frequency bands (*p* < 0.05; *t* test across site pairs) (Fig. 5a) (see the “Methods” section for details). This suggests that it is a locally generated low-frequency neural activity that coordinates the timing of spikes.

Although the main effect of attentional modulation of SPC is observed in the frequency bands with the centers between 3 and 8 Hz, there are several frequency bands higher than this range that still show small differences of SPC between the two attention conditions (frequency bands with centers of 9 and 15 Hz) (Fig. 3). However, the effect in these bands is only marginally significant (0.01 < *p* < 0.05 paired *t* test, corrected for multiple comparisons). Thus, if we limit the analysis to sites that have a higher number of trials, the effect in these frequency bands diminishes, while the main modulation in the frequency bands with centers of 3–8 Hz does not change appreciably (Fig. 5b). This suggests that the small difference between the attended and unattended SPC indices at the frequency bands above the 8 Hz band is mostly due to noise.

### Locking phase is not modulated by attention

**Fig. 6:**
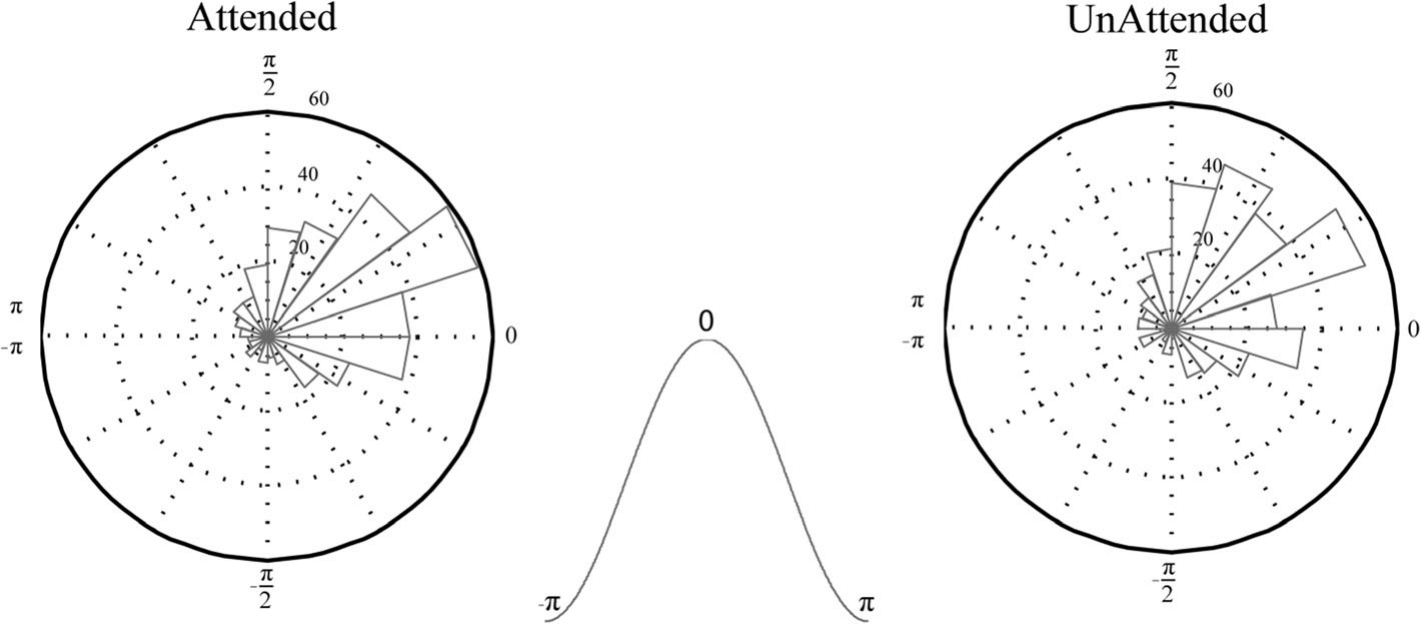
Histogram of locking phases for trials in which the target RDP was inside the RF (attended) or outside the RF (unattended). The inset in the middle indicates the phase values in a cycle

As shown in Fig. 3, MUA in MT is significantly locked to a specific phase within the 3–17 Hz frequency bands in each site pair, and this is true for both trials with the target inside or outside the RF. To determine whether the phases corresponding to different site pairs were similar, we extracted the locking phases for the frequency band 2–6 Hz in which the SPC strength’s modulation is the strongest (Fig. 3) (see the “Methods” section for details). Figure 6 shows the histogram of phases that each site pair pointed to for both attention conditions. The distribution of phases across different pairs differs significantly from the uniform distribution in both conditions (*p* < 10^−45^ for both conditions; von Mises test). The mean locking phase is 0.57 and 0.69 rad for attended and unattended conditions, respectively, and there is no statistically significant difference between them (*p* > 0.1 Watson-Williams test). This suggests that MUA spikes tend to occur mostly at a unique phase of theta LFPs independent of the attention condition.

**Fig. 7:**
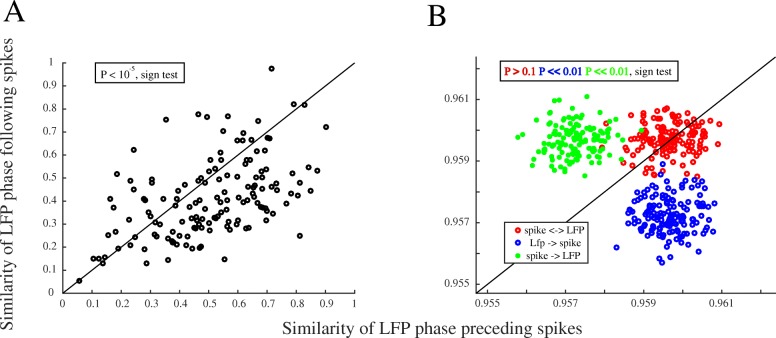
Directional interaction of LFP phase and spikes. **a** Similarity of the LFP phase before vs. after spikes. *x*- and *y*-axes show the similarity of instantaneous phases of the 1–10 Hz LFP preceding and following the spikes of each neuron, respectively. Each dot shows the phase similarity measure for one site from the three animals. **b** Simulation of the phase similarity for the LFP preceding vs. following spikes for virtual recording sites, under three conditions; similar phase similarity preceding and following spikes (shown in red), phase similarity preceding spikes higher than following spikes (in blue), phase similarity following spikes higher than preceding spikes (in green). This simulation indicates that our comparison of the phase similarity between intervals preceding and following spikes is not biased by the different distributions of STL’s phases relative to the spike

It was shown before that attention suppresses inter-neuronal synchrony at low frequencies [26, 27]. However, the neural mechanism of this modulation has remained elusive. Here, we hypothesize that the brain uses SPC to control inter-neuronal synchrony, i.e., by de-coupling neural spikes from the population-level synaptic activity (reflected by the LFP phase), neurons are free to fire independently of each other. We showed here that attention decreases SPC; however, it may be that LFP and SPC are epiphenomena reflecting the spikes and the inter-neuronal synchrony, respectively. Here, we test if the phase of the synaptic activity (reflected by LFP) or the spikes influences the other at a larger degree. Using an approach suggested previously [40], we calculated the angular similarity of the LFP phase preceding the spikes and compared it to that of the LFP phase following the spikes. In case it is the phase of the LFP-linked synaptic activity that has a larger influence on the occurrence of spikes, rather than the spikes being more influential on the LFP phase, we expect a more stable LFP pattern before spikes compared to after spikes. This means that for the phase of the LFP-linked synaptic activity to evoke the spikes, there should be a smaller phase variability preceding spikes rather than following spikes. We band pass filtered the LFPs between 1 and 10 Hz and calculated the spike-triggered LFP (STL) for 200 ms time windows centered on spike times (with the spikes and LFP coming from the same recording site, to avoid potential variabilities of time lags between electrodes’ signals induced by the traveling of LFP waves). Next, we calculated the instantaneous phases by applying the Hilbert transform on each trial’s STL curve in a given recording site and measured the phase similarity across trials for each STL time point by measuring the length of the vector given by cross-trial circular averaging. Comparison of the phase similarity preceding spikes (corresponding to the first half of STL) to the phase similarity following spikes showed a significantly higher phase similarity for LFPs preceding, rather than following spikes (*p* < 10^−5^; sign test across sites from all animals) (Fig. 7a). To further examine if it is the asymmetry of phases (different proportions of flat/steep parts) between the intervals preceding or following spikes that causes the difference in phase similarity of the two intervals, the following simulations were conducted: (1) We generated an artificial STL corresponding to each trial (*N* = 50) in a given site (as a simple single frequency (3.5 Hz) 200 ms sinusoid function), where the phase instantaneous to the spike (the STL’s value in the middle time point) equaled 0.55 rad (matching our empirical data in Fig. 6). Correspondingly, at this step, the STLs across all trials and sites were equal. (2) The instantaneous phases were calculated for the STLs and added by a randomly generated white noise signal ([0 1]) with the same length as the original STL, after which an additional random noise (with a 3% magnitude of the initial noise) was independently generated and summed with (a) each of the instantaneous phases preceding a spike, (b) following a spike, and (c) none of the intervals, independently in each trial, to create a higher phase variation within (a) the interval preceding the spikes, (b) the interval following the spikes, or (c) maintain the phase variation between the intervals equal. (3) We finally calculated the phase similarity using the same procedure as with our empirical data across trials for each simulated site, separated for the intervals preceding and following the spike. The computed phase similarity for each site is illustrated in Fig. 7b, with each of the noise conditions shown in a different color. This clearly shows that a similar trial-by-trial phase variation between the intervals following and preceding spikes causes also an equal phase similarity across the two intervals, irrespective of the asymmetry between the two intervals’ phases. In line with our hypothesis, this suggests that it is the phase of the LFP-linked synaptic activity that is more influential on the occurrence of spikes, rather than the spikes influencing this phase (equivalent to an epiphenomenal account of the phase). Therefore, the suppression of SPC by attention is an indication of a potential role of this LFP-linked synaptic activity in determining the inter-neuronal correlation.

## Discussion

We investigated whether action potentials are locked to the phase of low-frequency LFPs in the extrastriate macaque visual cortical area MT and if spatial attention modulates this potential locking. Our data show that neuronal spike times in the MT are preferentially aligned to the falling phases of the low-frequency LFP phases and that this alignment is reduced for responses to stimuli within the spotlight of spatial attention.

This spike-phase coupling (SPC) is consistent with the previous reports on the locking of MUA timing to the phase of ongoing low-frequency LFP in a number of different cortical regions of macaque, such as the hippocampus [10], prefrontal cortex [9], and especially visual areas including V1 [7, 22] and V4 [24]. The preferred phase of the field potential at which the MUA occurs differs across visual areas. In the primary visual area V1, MUA was shown to occur preferably at troughs [7, 22], while in the extrastriate cortex such as visual area V4, the preferred phase is somewhat earlier in the falling phase [24]. This similarity between the V4 and MT, areas at comparable levels of the cortical processing hierarchy suggest that the exact spike-phase alignment might depend on the level of a given area within a cortical processing pathway.

Our data show that the alignment of spikes to phases of the 3–8 Hz frequency bands decreases with attention. This is in line with earlier reports (e.g., [20, 39]), which found that neurons in the visual cortex V4 lose their coupling to low frequencies (< 10 Hz) with spatial attention. They calculated the magnitude of locking between spikes and field potentials by quantifying the *spike-field coherence* (SFC [41]). The SFC however does not exclusively reflect the locking of spikes to oscillation phases; rather, it computes a value between 0 and 1 for a given frequency, by comparing the power of the frequency in the spike-triggered average (STA) and the mean power of the frequency in each of the LFP segments that make up the STA. This measure however does not rule out the bias to the power of a given frequency at different times, i.e., a spike that occurs during a high amplitude period of the LFP will have a greater influence on the value of the SFC than a spike that has occurred during a low-amplitude LFP period. Therefore, the SFC reflects the locking of spikes to both the phase and amplitude of LFPs, rather than only the phase. The SPC metric solely however considers the phases of the LFP that the spikes have occurred at and thereby directly quantifies the coupling of spikes and LFP phases.

The decrease of low-frequency SFC by attention has been shown to originate in the neurons of deep rather than superficial layers [19] in area V4. We could not look for this difference in our data due to the lack of layer information in our recordings. Also, it is not clear to what extent the stimulus onset may couple the spikes to the low-frequency LFP. In spite of our observation that SPC differs across attention conditions (irrespective of visual stimulation), and a previous report showing in V4, that SFC exists also during periods of no visual stimulation [20], it still remains a question for future studies, how visual stimulation could modulate the coupling of spikes to LFP phase either in V4 or MT.

Not only the spikes are locked to the phase of low frequencies, but the power of high-frequency oscillations is also coupled to the phase of low frequencies across many cortical regions in humans, monkeys, and rats [7, 42, 43], an effect known as phase-amplitude coupling (PAC). On the other hand, spike rate and high-frequency power have been reported to be positively correlated with each other in the rat hippocampus [5] and monkey visual cortex [7], including the area MT [6], but see [44] for negative correlations). Therefore, our finding is consistent with our previous observation of an attentional suppression of PAC in the area MT [38]. Similarly, given the suggestion [39] that attention decreases the locking of spikes to the low-frequency phase in the area V4, we predict that as in the MT, attention decreases PAC in the area V4 as well. It remains a question for future studies to what degree SPC influences PAC and vice versa.

**Fig. 8:**
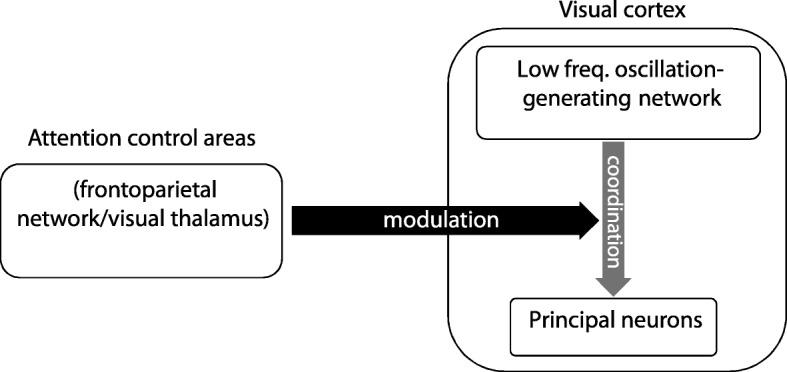
Schematic mechanism of attention’s modulation of SPC. This scheme shows how attention may determine the low-frequency synchrony between neurons, via modulating their coupling to an intra-regional subnetwork that generates low-frequency activity (potentially consisted of O-LM inter-neurons). This subnetwork’s local connection to the principal cells might be regulated by the feedback as well as neuromodulatory projections that are carrying the attention signal to the visual cortex (here, MT)

Cohen and Maunsell showed that visual attention decreases inter-neuronal correlations in the visual cortex, i.e., the correlation between the firing rate of neurons recorded simultaneously decreases when a monkey attends to a stimulus [27]. Similarly, Mitchell et al. found that simultaneously recorded neurons in the area V4 of monkeys reduce their correlated low-frequency fluctuations in firing rate when attention is directed into the RF [26]. As suggested previously, this decrease in inter-neuronal correlations can lead to the increase of neural discrimination and improvement of behavior, which are both benefits of attention [38, 45, 46]. Given the tight link between inter-neuronal synchrony and LFP [47], one potential mechanism underlying this decrease in inter-neuronal correlations might be a decoupling of neuronal spike times to the phase of the low-frequency LFP-linked synaptic activity. Since our analytical results suggest a significantly more stable pattern of LFP phase preceding, rather than following spikes, and that the low-frequency LFP is spread across plenty of neighboring cortical columns [8], the low-frequency component of the LFP-linked population synaptic activity could be a candidate for controlling the synchrony of neurons in different columns. One potential mechanism for this is that a regulatory network generates the low-frequency modulatory oscillatory activity, governing the activity of the network of principal cells [48] (reflected by the coupling of single neuron spikes to the low-frequency LFP). The degree to which the regulatory network influences the timing of the principal cells’ activity is presumably determined by the strength of the synaptic inputs the regulatory network projects to the principal cells. Attention signal could control the two networks’ coupling by inhibiting these synapses (Fig. 8). Such a low-frequency generating network has been found before in the hippocampus, predominated by O-LM inter-neurons (see [48] for a review). These cells are extensively projecting to inter-neurons and principal cells (while connections in the opposite direction are sparse), activating them in an oscillatory regime. The segregation of the O-LM network from that of the pyramidal-basket cell recurrent circuitry (extended longitudinally vs. transversally, respectively relative to cortical columns) further supports the existence of a low-frequency generating network and its causal influence on principal cells [49]. In addition to controlling the synchrony between neurons, such a low-frequency oscillation generator may play a role in communication with the downstream areas [24], as well as be used as a temporal window for local circuit computation and information representation [50, 51]. Identification of the cell types constituting the low-frequency generating neural network and how segregated they are within the visual cortex local circuitry, as well as the brain region/mechanism via which attention modulates this network’s projection on the principal cells, needs to be addressed by future studies. There are different cortical and subcortical regions known to be involved in producing neural signatures of attention in the visual cortex. Among the cortical areas, FEF and LIP have been reported to induce attentional effects in the extrastriate visual areas [52, 53], and in the subcortical regions, the pulvinar has been observed to mediate attentional effects on the visual cortex [54]. These areas, as well as the neuromodulatory system [55], are prime candidates for the attentional modulation of SPC in areas of the visual cortex.

## Conclusions

In summary, our data show that spike-LFP phase coupling exists along the dorsal visual pathway and that selective attention loosens this coupling. We finally suggest that attention harnesses this spike-phase coupling to de-synchronize neurons and thereby enhance the neural representation of the attended stimuli.
